# Supplementary material for: ConSolv: Solvent-Conditional Machine Learning Implicit Solvent Potential
Source: J Chem Theory Comput. 2026 Oct 12;22(20):10855–69. doi: 10.1021/acs.jctc.6c01253 (PMC13718550; doi:10.1021/acs.jctc.6c01253)
Supplement: Supplementary file 1 [file ct6c01253_si_001.pdf]

Supporting Information: ConSolv: Solvent-Conditional Machine Learning Implicit Solvent Potential

Linying Zhang[1] and Julija Zavadlav*[1,2]

[1]Multiscale Modeling of Fluid Materials, Department of Engineering Physics and Computation, TUM School of Engineering and Design, Technical University of Munich, Germany
[2]Atomistic Modeling Center (AMC), Munich Data Science Institute (MDSI), Technical University of Munich, Germany

*Email: julija.zavadlav@tum.de

S1 Training details

Table S1: Hyperparameter settings used in this work.

**MACE architecture hyperparameters**			
**Hyperparameter**	**Value**		
Interaction layers	2		
Cutoff length	4 Å		
Hidden irreps	`64x0e + 32x1o`		
Max L	3		
Atom embedding dimension	64		
Solvent embedding dimension	3		
**Solvent embedding block hyperparameters**			
**Hyperparameter (Node level)**	**Value**		
Number of attention heads n	8		
Solvent feature projection dimension p	64		
Key size of each head d	64		
Value size of each head d_v	64		
**Hyperparameter (Edge level)**	**Value**		
Hidden layer number	1		
Hidden layer dimension	64		
Activation function	SiLU		
**Numerical optimization hyperparameters**			
**Hyperparameter**	**Stage 1.1** (U_{vac})	**Stage 1.2** (U_{vac})	**Stage 2** (U_{sol})
Method	Adam	Adam	Adam
Initial learning rate	1×10^{-2}	2.5×10^{-4}	5×10^{-5}
Learning rate decay	1	1	10^{-2}
Number of epochs	50	50	50
Batch size	200	200	5

S2 Descriptors

Table S2: List of selected molecular descriptors.

Descriptor	Short Name	Description
Kappa1	Shape Index (Linear-Branched)	Kier & Hall molecular shape index (1st order).
MaxPartialCharge	Most Positive Atom Charge	Maximum atomic partial charge in the molecule.
MinEStateIndex	Lowest Electrotopo. Value	Minimum electrotopological state (E-State) value.
MinPartialCharge	Most Negative Atom Charge	Minimum atomic partial charge in the molecule.
ExactMolWt	Monoisotopic Mass	Exact molecular weight based on atomic masses.
qed	Drug-likeness Score	Quantitative Estimate of Drug-likeness (QED score).
MolLogP	Octanol–Water Partition Coefficient	Octanol-water partition coefficient (logP, Wildman-Crippen).
MolMR	Light Refraction Capacity	Molecular refractivity (Wildman-Crippen).
MolWt	Average Molecular Mass	Average molecular weight.
NumRotatableBonds	Flexible Bond Count	Number of rotatable bonds.
PEOE_VSA1	Surface Area by Charge (Bin 1)	Van der Waals surface area contribution bin (partial charge).
SMR_VSA1	Surface Area by Refractivity (Bin 1)	VSA contribution bin (molar refractivity).
SlogP_VSA1	Surface Area by LogP (Bin 1)	VSA contribution bin (logP).
VSA_EState1	Surface Area by E-State (Bin 1)	VSA contribution bin (E-State index).
fr_Al_OH	Aliphatic -OH Count	Count of aliphatic hydroxyl groups.

S3 Alternative models

S3.1 Output-based solvent embedding model

To demonstrate the effectiveness of our proposed solvent embedding block, we implemented an alternative model where the solvent information is directly integrated into the output of the MACE vacuum model. Specifically, we added the solvent-descriptor-based attention mechanism to the atomic energy contributions predicted by the MACE vacuum model before summing them up to obtain the total energy. The attention block is calculated as described in Eq. 4, where the input query is replaced with the atomic energy contributions:

$$\hat{U}_i = U_i + \mathrm{MultiHead}(U_i, F_k, F_k), \tag{1}$$

where U_i is the atomic energy contribution at the output layer. Meanwhile, the edge features are not augmented.

S3.2 Trainable solvent embedding vector

The trainable solvent embedding is tested with both the attention mechanism and simple concatenation. For each solvent k, an embedding vector $\mathbf{S}_k \in \mathbb{R}^{N_d}$ is indexed from a lookup table and randomly

initialized, where $N_d = 15$. For the attention mechanism, the procedure is identical to the descriptor-based approach: $\mathbf{S}_k$ is first projected to a feature matrix $F_k \in \mathbb{R}^{N_d \times p}$ via a linear layer, which then serves as both key and value in the cross-attention computation following Eq. 4. The sole difference from the descriptor-based approach is that $\mathbf{S}_k$ is a free parameter optimized during training rather than a fixed vector of physicochemical descriptors.

For the simple concatenation approach, the solvent embedding vector $\mathbf{S}_k$ is concatenated with the node embedding $\mathbf{h}_i$, followed by a linear transformation and a sigmoid-weighted linear unit (SiLU) activation function. The updated node embedding $\hat{\mathbf{h}}_i$ is computed as:

$$\hat{\mathbf{h}}_i = \mathbf{h}_i + \mathrm{SiLU}(\mathrm{Linear}(\mathrm{Concat}(\mathbf{S}_k, \mathbf{h}_i))), \tag{2}$$

where $\mathrm{Concat}(\mathbf{S}_k, \mathbf{h}_i)$ denotes the concatenation of the solvent embedding vector $\mathbf{S}_k$ and the node embedding $\mathbf{h}_i$, $\mathrm{Linear}(\cdot)$ represents a learnable linear transformation, and $\mathrm{SiLU}(\cdot)$ denotes the activation function. Hyperparameters for the solvent embedding block are reported in Table S1.

S4 Fluorohydrin J-coupling constant evaluation

Following the methodology and standard reference values described by Morado *et al.* [1], the conformers of γ-fluorohydrins are defined based on the rotation of their threefold torsional barriers: χ (C-C-C-O), ϕ (F-C-C-C), and ψ (C-C-O-H). Each dihedral angle is categorized into one of three rotamer states:

- $0° \leq \chi, \phi, \psi < 120° \Rightarrow \mathrm{g}^+$
- $120° \leq \chi, \phi, \psi < 240° \Rightarrow \mathrm{t}$
- $-120° \leq \chi, \phi, \psi < 0° \Rightarrow \mathrm{g}^-$

By clustering the individual frames of the molecular dynamics (MD) trajectories according to these torsional boundaries, we obtain the simulated conformer populations (P_q^ν) for the evaluated models. To compute the macroscopic J-coupling constants, we utilize the reference theoretical J-coupling values ($J_{\lambda,q}$) for each conformer state q, which were previously calculated at B97-2/pcJ-2 with PCM on geometries optimized at ωB97X/6-311++G(2d,p)/PCM by Morado *et al.* [1]. The final ensemble-averaged J-coupling constant J_λ^ν for a given model ν is then evaluated as the weighted average over all conformers based on their respective populations at 298.15 K:

$$J_\lambda^\nu = \sum_{q=1}^{N_{\mathrm{conf}}} P_q^\nu J_{\lambda,q} \tag{3}$$

where N_{conf} denotes the total number of identified conformers.

S5 Additional results

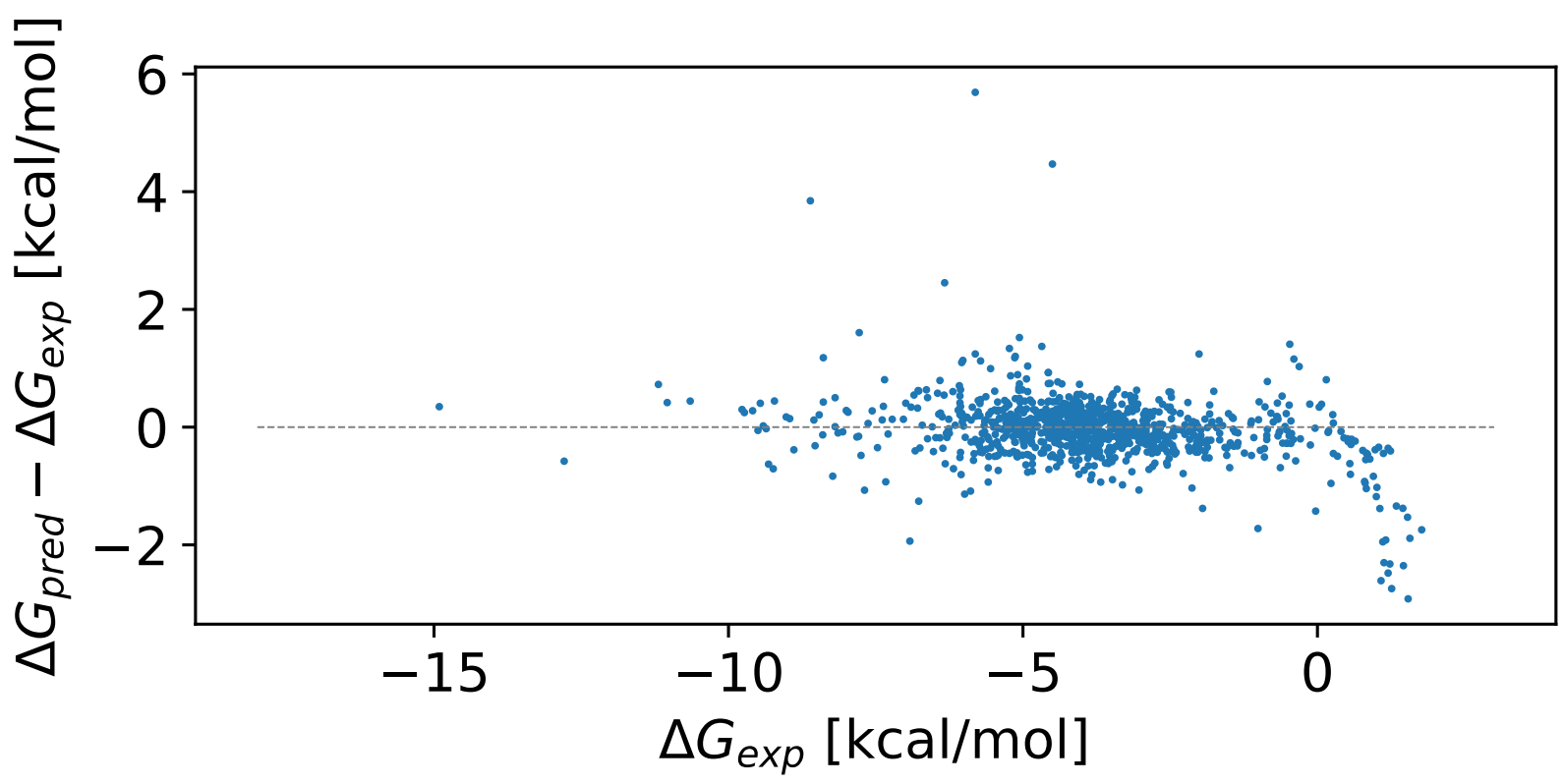

Figure S1: Error of the solvation free energy prediction for each data point in the test set.

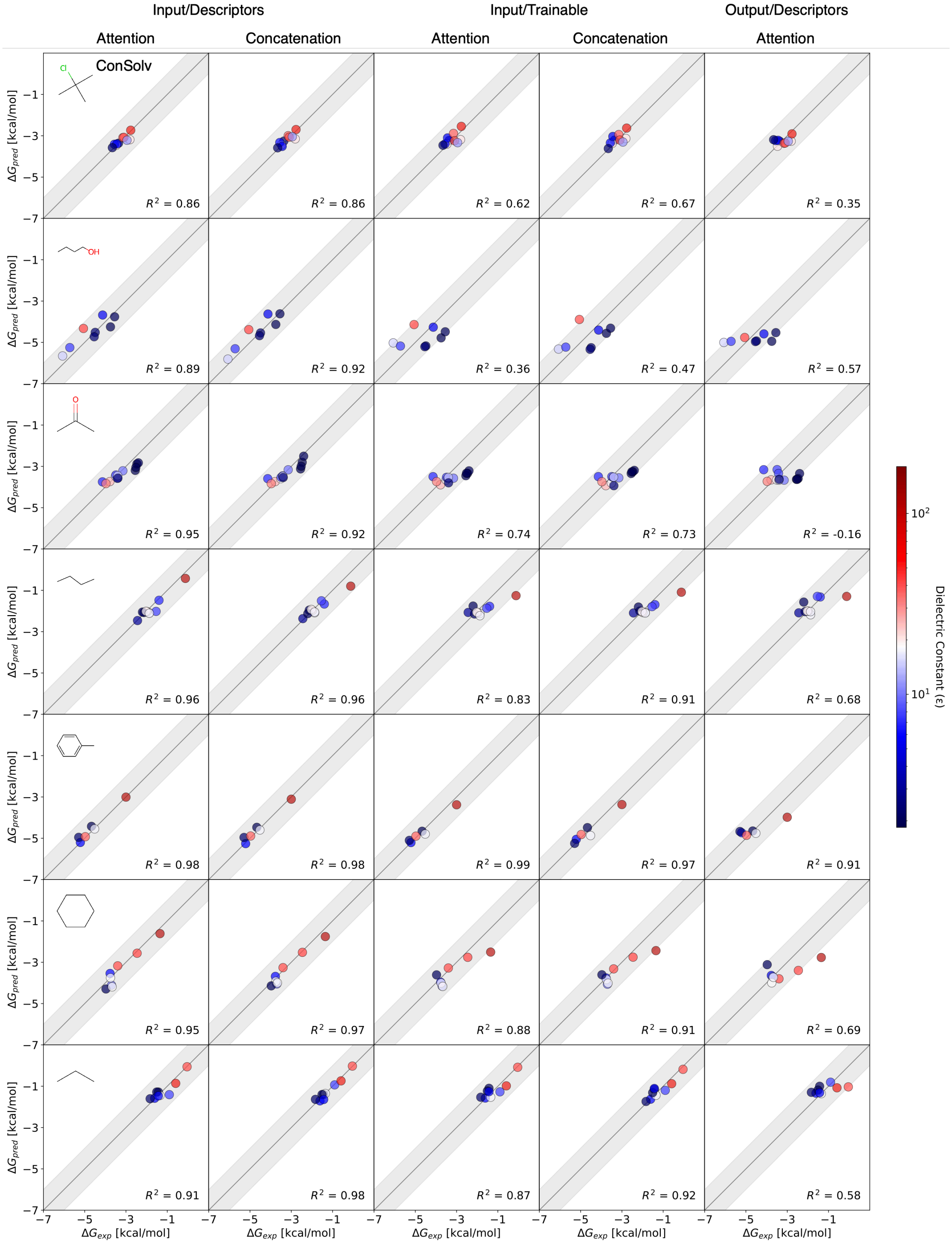

Figure S2: Comparison of the solvation free energy prediction.

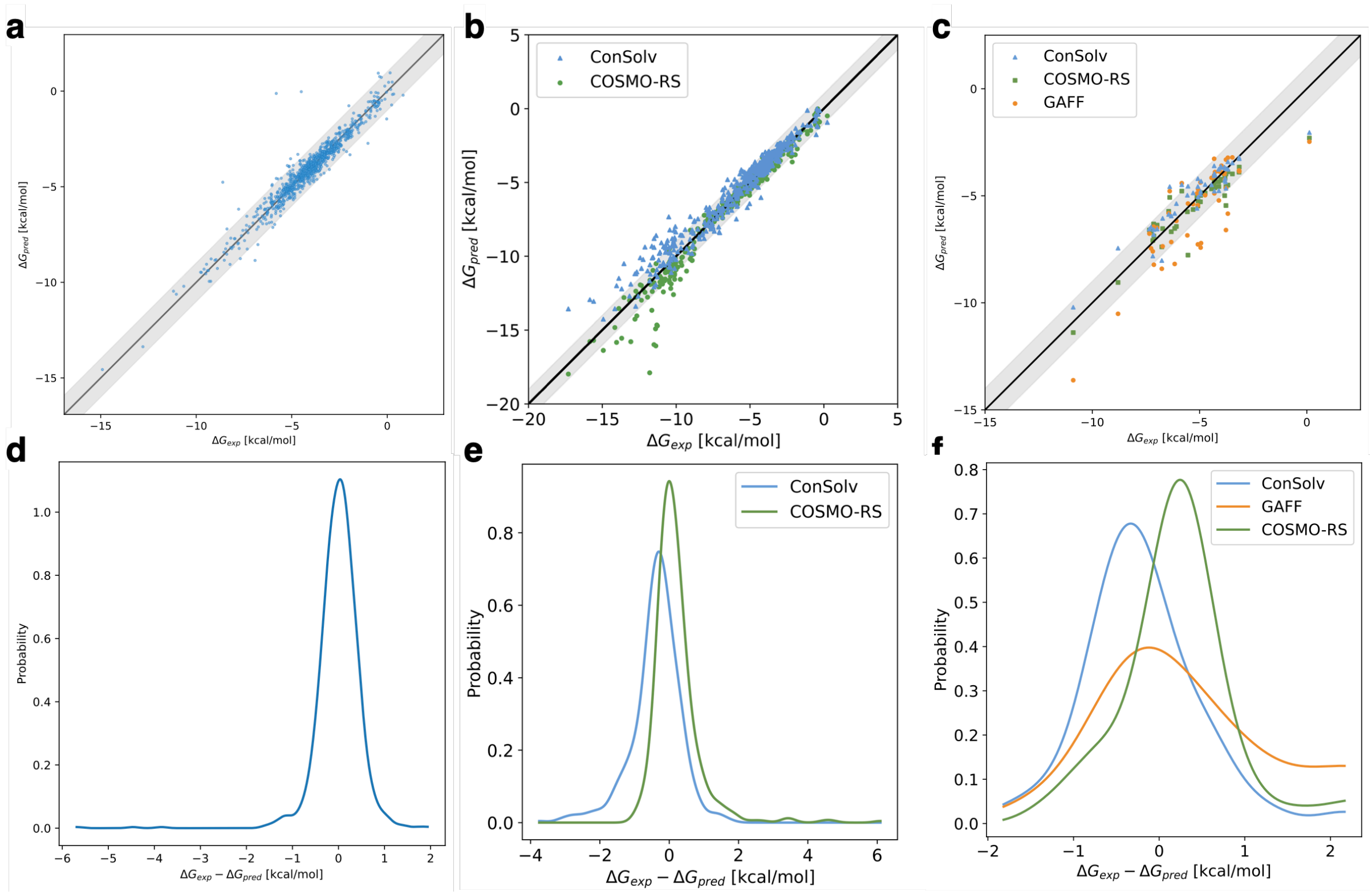

Figure S3: **Predictions on test set.** (a) Pairplot comparison between ConSolv and experimental values on Solv@TUM test set; (b) Pairplot comparison between ConSolv and COSMO-RS on CombiSolv [2]; (c) Pairplot comparison between ConSolv, COSMO-RS and GAFF on the Zhang *et al.* [3] benchmark; (d) Error distribution of ConSolv on the corresponding test set as shown in (a); (e) Error distribution of ConSolv and COSMO-RS on CombiSolv [2]; (f) Error distribution of ConSolv, COSMO-RS and GAFF on the Zhang *et al.* [3] benchmark.

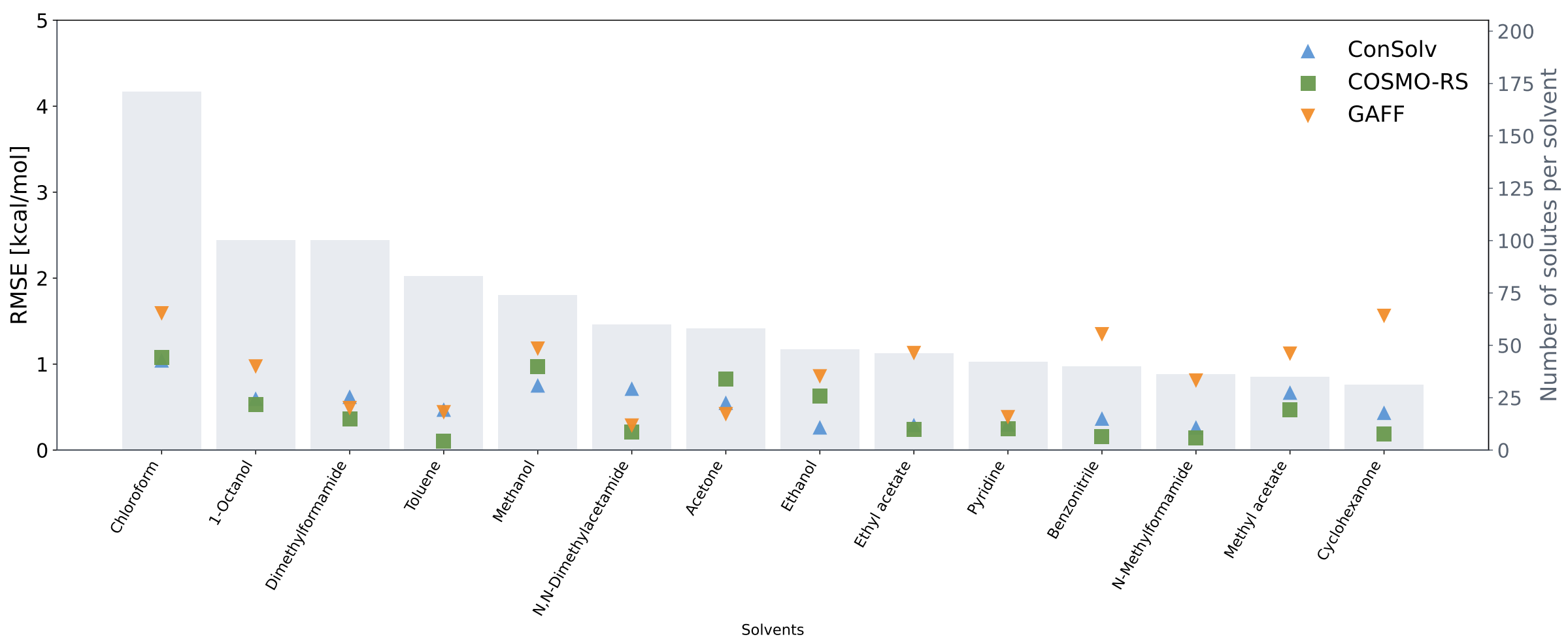

Figure S4: Per-solvent root mean squared error (RMSE, kcal/mol) for GAFF (orange circles), COSMO-RS (green squares), and ConSolv (blue triangles) on the Zhang *et al.* [3] benchmark.

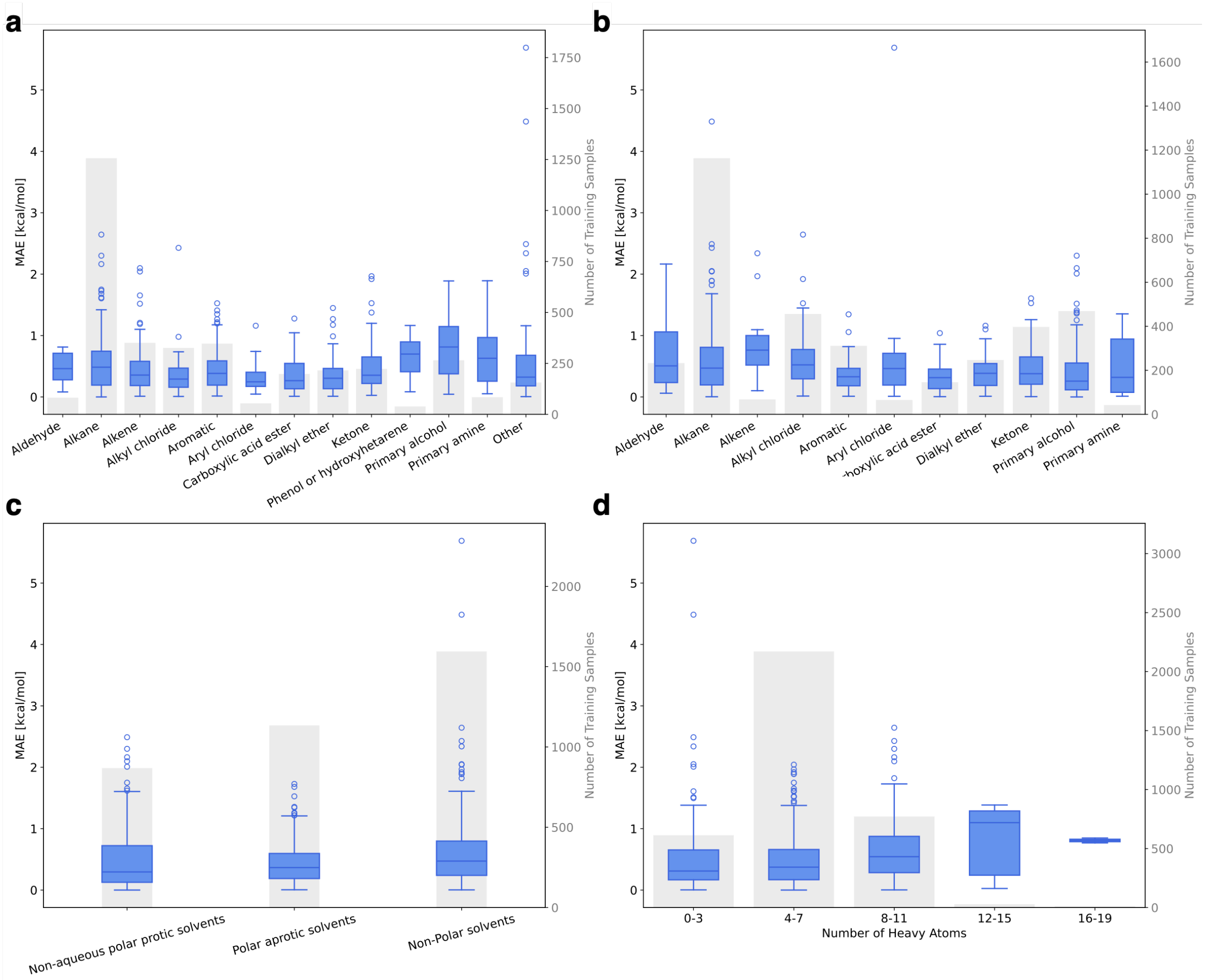

Figure S5: **Error analysis of ConSolv predictions on the test set.** (a) Grouped error based on different solvent functional groups; (b) Grouped error based on different solute functional groups; (c) Grouped error based on the category of solvent; (d) Grouped error based on the number of heavy atoms in the solute.

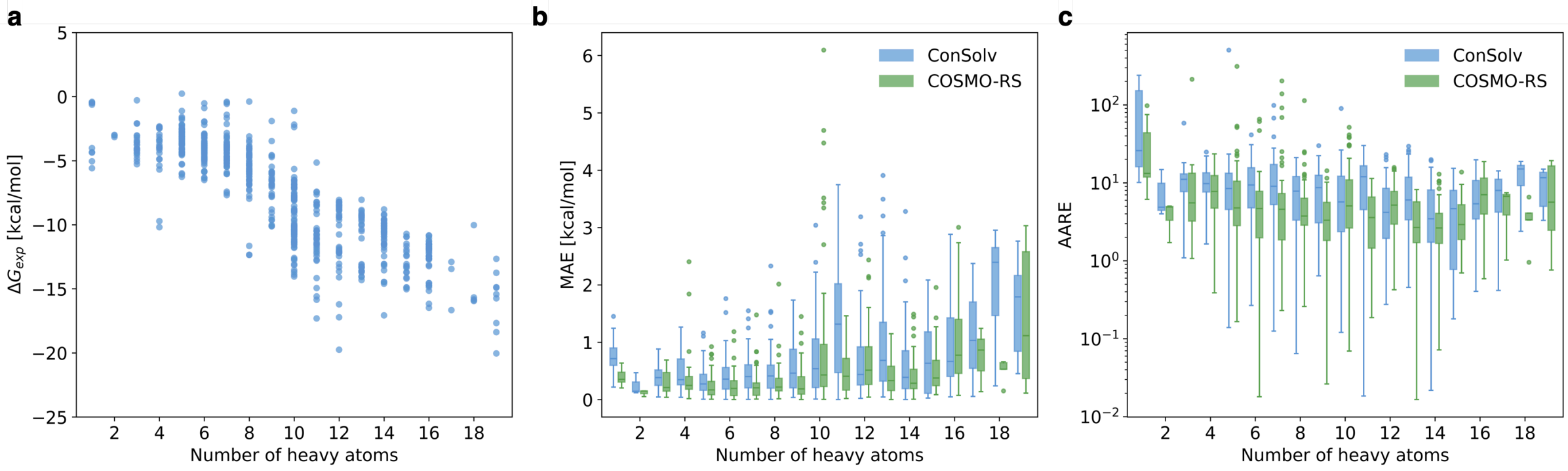

Figure S6: **Error analysis of ConSolv predictions on CombiSolv [2].** (a) Distribution of experimental solvation free energies as a function of the number of heavy atoms in the solute; (b) mean absolute error (MAE) grouped by the number of heavy atoms in the solute; (c) average absolute relative error (AARE) grouped by the number of heavy atoms in the solute.

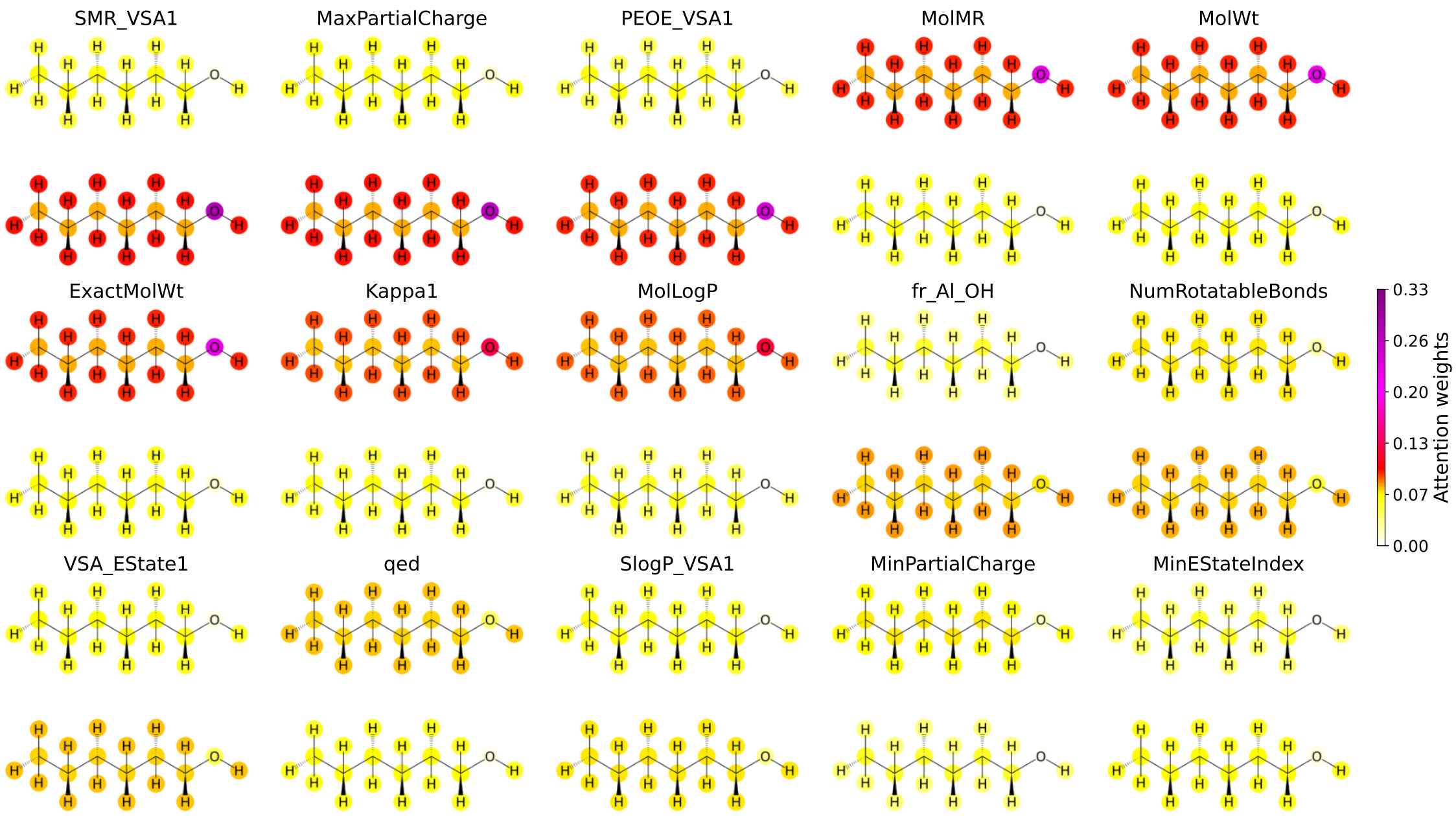

Figure S7: Per-atom attention weights (head$_7$) for 1-hexanol solute molecule and solvent descriptors of methanol (top, $\varepsilon = 32.61$, polar) and 2,2,4-trimethylpentane (bottom, $\varepsilon = 1.93$, non-polar).

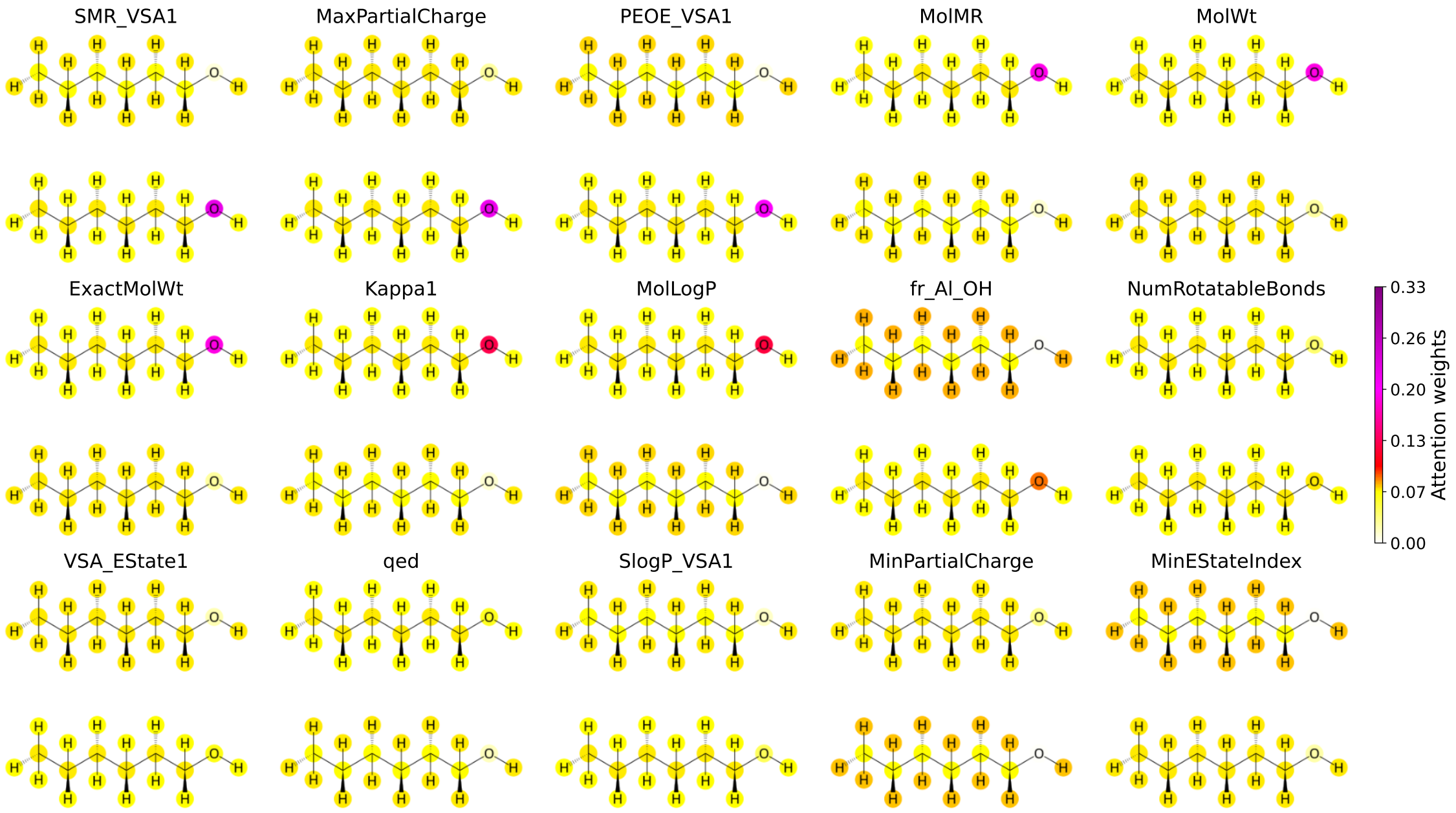

Figure S8: Per-atom attention weights ($head_2$) for 1-hexanol solute molecule and solvent descriptors of methanol (top, $\varepsilon = 32.61$, polar) and 2,2,4-trimethylpentane (bottom, $\varepsilon = 1.93$, non-polar).

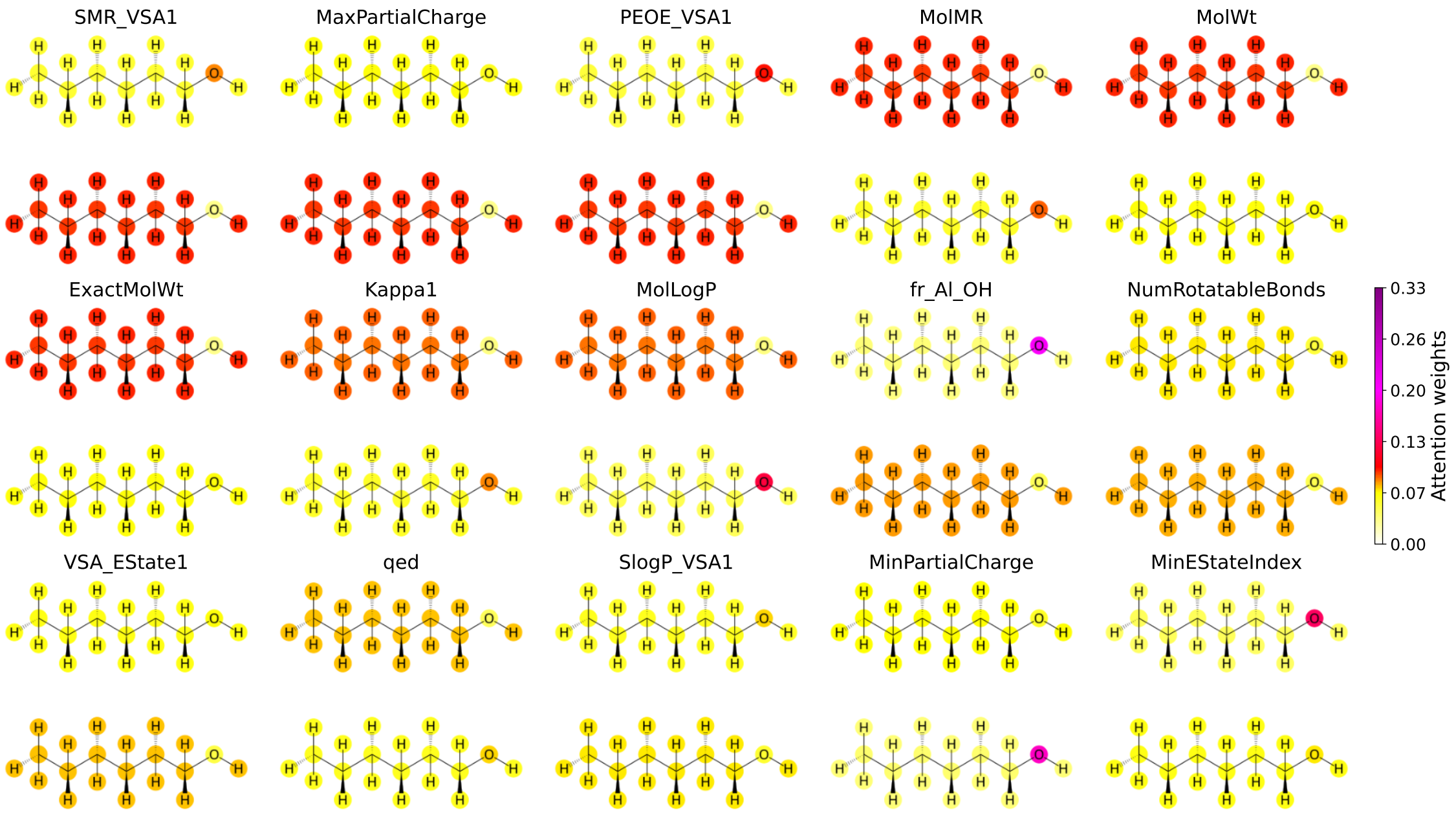

Figure S9: Per-atom attention weights ($head_5$) for 1-hexanol solute molecule and solvent descriptors of methanol (top, $\varepsilon = 32.61$, polar) and 2,2,4-trimethylpentane (bottom, $\varepsilon = 1.93$, non-polar).

S6 Fixed-coordinate Generalized Born reference calculation

The Generalized Born calculation was used only as a fixed-coordinate reference for the polar electrostatic response. No GB trajectory was propagated. Instead, for each solvent, the same 500 configurations sampled from the corresponding ConSolv production trajectory were evaluated with OpenMM 8.0 [4] GBn2 [5] using GAFF2 parameters [6], AM1-BCC atomic charges [7], and `mbondi3` Born radii [5]. The solute dielectric constant was 1.0, and the solvent dielectric constant, ε_s, was set to 110.5, 32.7, 8.1, 2.2, or 2.0 for formamide, methanol, 1-decanol, carbon tetrachloride, or n-decane, respectively.

For each ConSolv configuration $\mathbf{r}$, the polar reaction-field contribution was calculated as

$$\Delta U_{\mathrm{GBn2}}(\mathbf{r}) = U_{\mathrm{GAFF2+GBn2}}(\mathbf{r}) - U_{\mathrm{GAFF2,vacuum}}(\mathbf{r}). \tag{4}$$

This polar energy was decomposed into atomic self-energy and pair-interaction terms according to

$$\Delta U_{\mathrm{GBn2}}(\mathbf{r}) = \sum_i U_i^{\mathrm{self}}(\mathbf{r}) + \sum_{i<j} U_{ij}^{\mathrm{int}}(\mathbf{r}), \tag{5}$$

where U_i^{self} is the Born self-energy of atom i and U_{ij}^{int} is the polar pair interaction between atoms i and j. The plotted atomic contribution was defined as

$$G_i(\mathbf{r}) = U_i^{\mathrm{self}}(\mathbf{r}) + \frac{1}{2}\sum_{j\neq i} U_{ij}^{\mathrm{int}}(\mathbf{r}). \tag{6}$$

Thus, each atom receives its complete self-energy and one-half of every pair interaction in which it participates, and $\sum_i G_i(\mathbf{r}) = \Delta U_{\mathrm{GBn2}}(\mathbf{r})$. The reported maps are averages over the same 500 ConSolv configurations used for the learned-model analysis.

Only the polar electrostatic component was retained because a consistent nonpolar parameterization is not available for the tested non-aqueous solvents. The ACE/nonpolar term was therefore disabled, so cavity-formation and dispersion contributions are excluded. Accordingly, the GBn2 maps in Figure S10 are used only to compare qualitative spatial localization with the learned per-atom energy shifts, not their absolute magnitudes. Differences along the hydrocarbon chain can arise because these omitted nonpolar terms are important for the alkyl group [8, 9] and because the learned per-atom energies are a model-dependent decomposition of the total energy rather than uniquely defined physical observables.

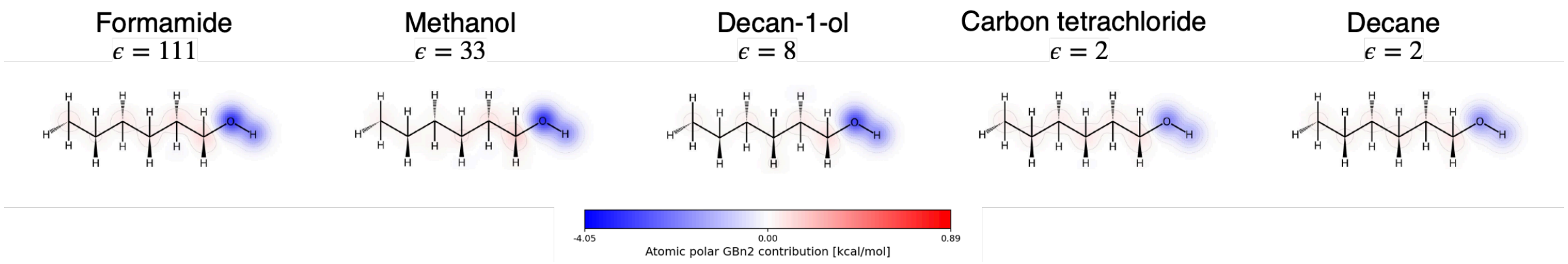

Figure S10: Trajectory-averaged atomic polar GBn2 contributions for 1-hexanol in formamide, methanol, 1-decanol, carbon tetrachloride, and n-decane. The labels give the solvent dielectric constants, ε_s. Blue and red denote stabilizing and destabilizing polar contributions, respectively.

References

(1) Morado, J.; Mortenson, P. N.; Nissink, J. W. M.; Essex, J. W.; Skylaris, C.-K. Does a machine-learned potential perform better than an optimally tuned traditional force field? A case study on fluorohydrins. *J. Chem. Inf. Model.* **2023**, *63*, 2810–2827.

(2) Vermeire, F. H.; Green, W. H. Transfer learning for solvation free energies: From quantum chemistry to experiments. *Chem. Eng. J.* **2021**, *418*, 129307.

(3) Zhang, J.; Tuguldur, B.; van der Spoel, D. Force field benchmark of organic liquids. 2. Gibbs energy of solvation. *J. Chem. Inf. Model.* **2015**, *55*, 1192–1201.

(4) Eastman, P.; Swails, J.; Chodera, J. D.; McGibbon, R. T.; Zhao, Y.; Beauchamp, K. A.; Wang, L.-P.; Simmonett, A. C.; Harrigan, M. P.; Stern, C. D.; Wiewiora, R. P.; Brooks, B. R.; Pande, V. S. OpenMM 7: Rapid Development of High Performance Algorithms for Molecular Dynamics. *PLoS Comput. Biol.* **2017**, *13*, e1005659, DOI: `10.1371/journal.pcbi.1005659`.

(5) Nguyen, H.; Roe, D. R.; Simmerling, C. Improved Generalized Born Solvent Model Parameters for Protein Simulations. *J. Chem. Theory Comput.* **2013**, *9*, 2020–2034, DOI: `10.1021/ct3010485`.

(6) Wang, J.; Wolf, R. M.; Caldwell, J. W.; Kollman, P. A.; Case, D. A. Development and Testing of a General Amber Force Field. *J. Comput. Chem.* **2004**, *25*, 1157–1174, DOI: `10.1002/jcc.20035`.

(7) Jakalian, A.; Jack, D. B.; Bayly, C. I. Fast, Efficient Generation of High-Quality Atomic Charges. AM1-BCC Model: II. Parameterization and Validation. *J. Comput. Chem.* **2002**, *23*, 1623–1641, DOI: `10.1002/jcc.10128`.

(8) Cramer, C. J.; Truhlar, D. G. Implicit Solvation Models: Equilibria, Structure, Spectra, and Dynamics. *Chem. Rev.* **1999**, *99*, 2161–2200, DOI: `10.1021/cr960149m`.

(9) Reichardt, C.; Welton, T., *Solvents and solvent effects in organic chemistry*, 4th ed.; Wiley-VCH: Weinheim, 2011, DOI: `10.1002/9783527632220`.
